# A novel mutation of the *MEN1* gene in a patient with multiple endocrine neoplasia type 1 and recurrent fibromyxoid sarcoma – a case report

**DOI:** 10.1186/s12881-020-01129-4

**Published:** 2020-09-29

**Authors:** Maja Radman, Tanja Milicevic

**Affiliations:** 1 Department of Endocrinology and Diabetology, University Hospital Centre Split, Soltanska 1, Split, Croatia; 2 University of Split, School of Medicine, Soltanska 2, Split, Croatia

**Keywords:** Multiple endocrine neoplasia type 1, Tumor-suppressing gene, Mutation, Low-grade fibromyxoid sarcoma

## Abstract

**Background:**

Multiple endocrine neoplasia type 1 (MEN1) syndrome is usually accompanied by endocrine tumors, but non-endocrine tumors can occur as well. However, the coexistence of MEN1 syndrome and malignant tumor such as low-grade fibromyxoid sarcoma has not been described in the literature. Moreover, the *MEN1* gene mutations have not been identified in patients with fibromyxoid sarcoma, so far.

**Case presentation:**

We present a patient with a long-year endocrine follow-up due to multiple endocrine tumors. During his lifespan, he has been surgically treated for pancreatic gastrinoma, parathyroid hyperplasia, atypical pulmonary carcinoid, various benign mesenchymal, and several skin tumors (basocellular tumor, lipomas, and fibromas) which raised a high clinical suspicion of MEN1 syndrome but the patient refused genetic testing. Recently, he developed a novel malignant tumor – recurrent low-grade fibromyxoid sarcoma of the trunk and extremities with multiple subsequent operations. The patient eventually accepted the genetic testing which proved him to be a carrier of a novel mutation in the *MEN1* gene.

**Conclusions:**

Unlike some other syndromes where a genetic mutation can predict clinical course, there is no genotype-phenotype correlation in MEN1 syndrome. Therefore, these patients require lifelong and multidisciplinary surveillance, not only for typical endocrine and benign non-endocrine tumors but also for diverse and even more malignant forms. The atypical clinical presentation should pose suspicion about new gene mutation and serve as a warning in the further follow-up.

## Background

Multiple endocrine neoplasia type 1 (MEN1) is a rare syndrome characterized by the development of endocrine tumors mainly of parathyroid, gastroenteropancreatic, and/or anterior pituitary origin. However, various non-endocrine, mostly benign tumors of the central nervous system, skin, smooth muscle, and other mesenchymal tumors can occur as well [1]. MEN1 syndrome is usually inherited in an autosomal-dominant manner, but a non-hereditary, sporadic occurrence is also possible. If inherited, a high degree of penetrance with apparent clinical manifestation is observable in almost all patients by the fifth decade of life [2]. Germline or somatic, mostly inactivating mutations of the coding region of the *MEN1* gene on the chromosome 11q13 result in a loss of a functional tumor-suppressor protein called menin. Its precise role is still under investigation, but it appears to have multiple roles in gene transcription and its dysfunction could lead to unregulated cell division and consequential tumor growth in susceptible tissues [3]. Fibromyxoid sarcoma is a common adult soft tissue sarcoma characterized by an infiltrative growth and a high local recurrence rate. To our knowledge, its association with MEN1 syndrome has not been previously described in the literature. Furthermore, the *MEN1* gene mutations have not been formerly reported in sarcomas but only occasionally in a benign smooth muscle tumors and lipomas [4]. Therefore, a risk of malignancy poses major complication for MEN1 patients. According to some authors, MEN1 patients have a 50% probability of death by the fifth decade whilst the cause of death in almost 50 to 70% of them is a malignant process or its sequelae [5–7]. The inability to predict tumor penetrance and the malignant transformation demands a life-long follow-up of MEN1 carriers to prevent morbidity and mortality [8]. Early diagnosis and treatment success are the main prognostic factors. We illustrate the problem with the following clinical case.

## Case presentation

**Fig. 1:**
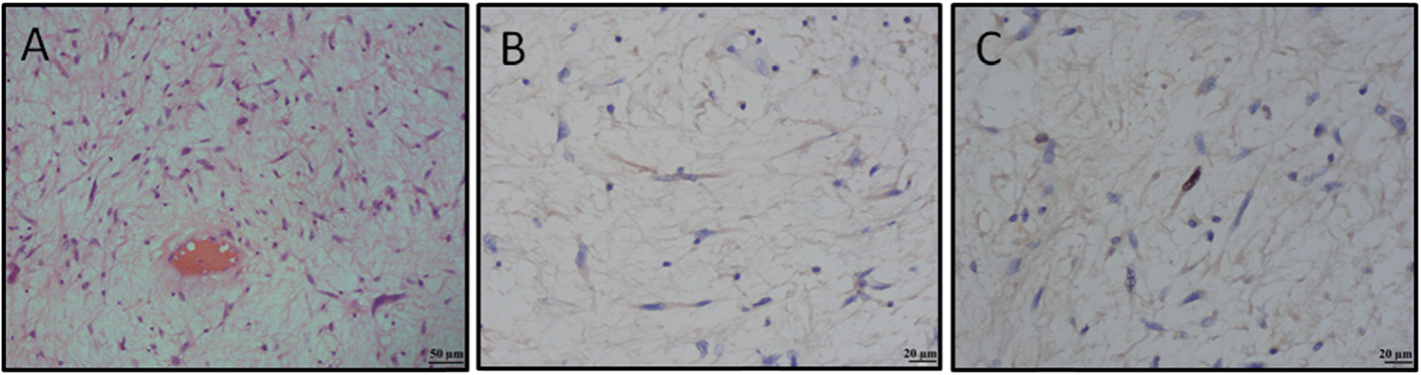
Microscopic (histologic) images of a low-grade fibromyxosarcoma. **a**) hypocellular neoplasm is composed of atypical fibroblasts and pseudo-lipoblasts embedded in a prominent myxoid matrix (H&E staining, 200x magnification), **b**) tumor cells stain positive for epithelial membrane antigen (EMA staining, 400x magnification), and **c**) proliferative activity in tumor cells is low (Ki-67 staining, 400x magnification)

**Fig. 2:**
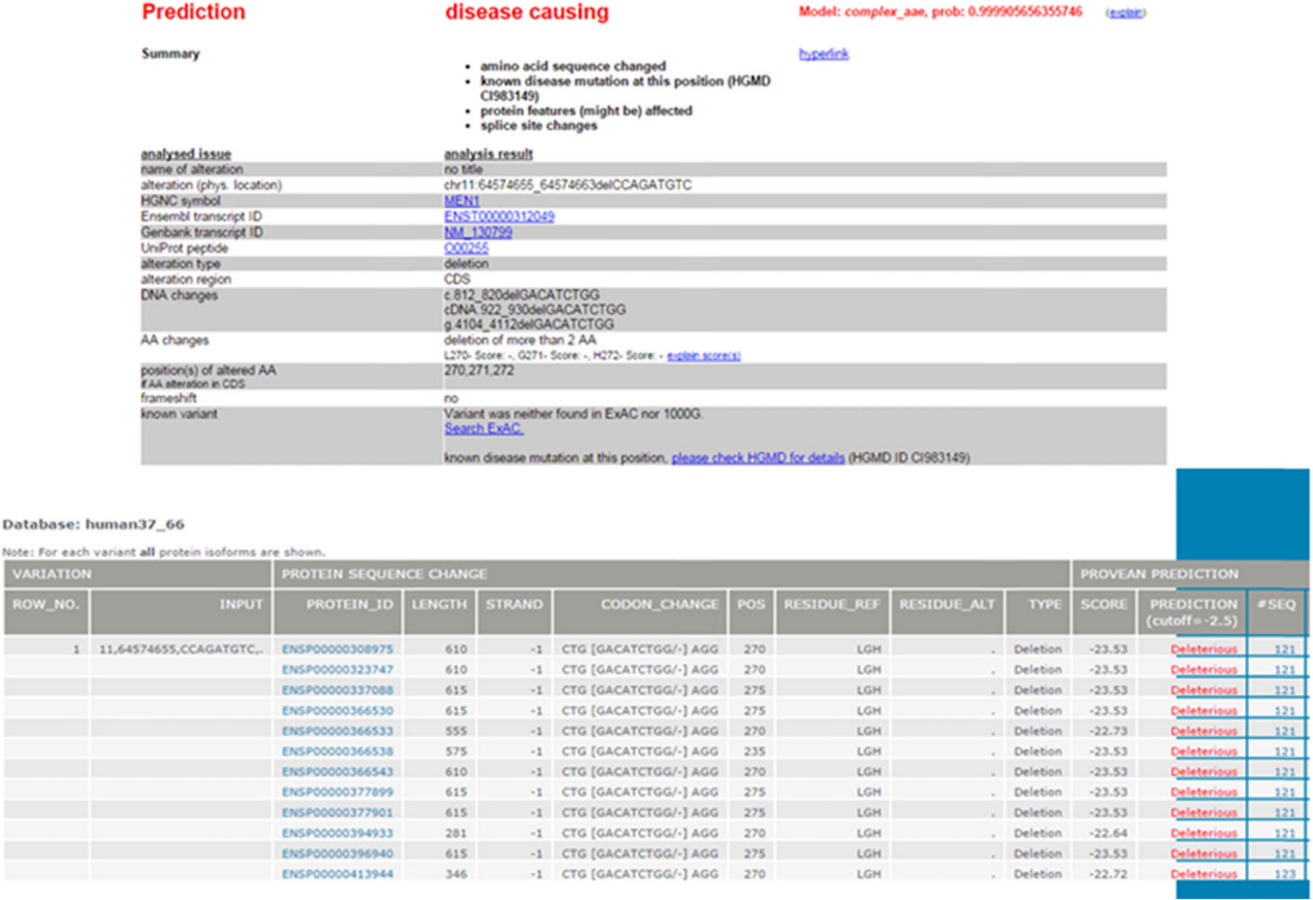
Analysis of the *MEN1* gene revealed a new mutation in exon 5 (variant c.812_820del, p.Gly271_Leu273del). The prediction of the new variant pathogenicity was performed with Mutation Taster (http://www.mutationtaster.org) and PROVEAN (http://provean.jcvi.org/index.php) software and revealed deleterious mutation

We report a case of a 57 year old man who was surgically treated for pancreatic gastrinoma and multiple gastric ulcers at the age of 32. Several years later he developed hypercalcemia and elevated parathyroid hormone levels which yielded further endocrine work-up. Early and late images of a pre-operative sestamibi scan showed multiple areas of increased tracer accumulation which consequently resulted in the extirpation of all four parathyroid glands and right thyroid lobectomy. The following pathohistological finding showed parathyroid hyperplasia of all four glands. Aforementioned medical data posed a high clinical suspicion of MEN1 syndrome but thereupon the patient lacked interest in genetic testing since he had no children. His parents and close relatives were generally healthy without apparent health issues such as endocrinopathies or tumors. During the routine follow-up at the age of 45, the patient was diagnosed with pulmonary neoplasia. Total excision was performed and the pulmonary neuroendocrine tumor (NET) grade 2 was established without any evidence of residual disease. A year later, a positron emission tomography–computed tomography scan showed an increased tracer uptake in the right thoracic paravertebral area. A successful tumor resection by video-assisted thoracoscopic surgery was conducted leading to schwannoma diagnosis. Later follow-up did not reveal any residual disease, but the patient continued medical check-up due to painless skin lumps on the limbs, torso and face. Multiple local operations revealed skin fibromas, lipomas and basocellular carcinoma of the face. However, at the age of 50, he underwent a surgical excision which showed a low-grade fibromyxoid sarcoma (Fig. 1). Over the next 4 years, the patient had multiple operations due to sarcoma recurrence at different sites. Furthermore, he was evaluated for mediastinal lymphadenopathy and diagnosed with NET metastasis (grade 2, Ki-67 18%). Adjuvant radiation therapy was applied and octreotide therapy was introduced which yielded good therapy tolerance and patient well-being on the last regular check-up. His medications included levothyroxine 50 mcg, calcitriol 0.25 mcg, calcium-carbonate 500 mg b.i.d. (with meals), insulin degludec 12 IU SC (before bedtime), insulin aspart 2–5 IU SC (before meals), and octreotide LAR 40 mg IM q4Weeks. The follow-up laboratory analyses show remarkable findings, while the imaging procedures do not reveal progression of the mediastinal lymphadenopathy. Ultimately, our patient affirmed genetic testing and turned out to be a carrier of a novel mutation variant of the *MEN1* gene. The sequencing of the *MEN1* gene included the whole coding region of the gene. Analysis of the *MEN1* gene coding region revealed a heterozygous mutation (variant c.812_820del, p.Gly271_Leu273del) in the exon 5. For the identification of mutations in the coding region of this gene, Applied Biosystems 3130xl Genetic analyzer and BigDye® Terminator v3.1 Cycle Sequencing Kit were used. Pathogenicity of identified mutation was verified in reference databases for mutations related to MEN1 syndrome (http://www.umd.be/MEN1/). The datasets generated and analysed during the current study are available in the GenBank and ENSEMBL database. The prediction of the new variant pathogenicity was performed with Mutation Taster (http://www.mutationtaster.org) and PROVEAN (http://provean.jcvi.org/index.php) software (Fig. 2).

## Discussion and conclusions

To date, more than 1300 mutations have been detected in the *MEN1* gene region. Our patient is a carrier of a novel mutation of the *MEN1* gene that has not been previously described in the literature. However, the exact inheritance pattern in our patient is unknown since his parents were not willing to undergo genetic testing. As far as we know, none of them or close relatives had developed apparent endocrinopathies or tumors during their lifetime. Even though the patient presented with characteristic MEN1 syndrome components earlier in his lifetime, a novel mutation and a negative family history made us to presume that he is rather a carrier of sporadic than hereditary mutation. Furthermore, except typical endocrine tumors and benign mesenchymal tumors that are common in MEN1 syndrome, our patient developed a recurrent low-grade fibromyxoid sarcoma. Analysing genetic and epigenetic profile of fibromyxoid sarcoma samples, Ogura et al. have found frequent alterations in p53 signalling, cell cycle checkpoint genes, and other driver genes such as *ATRX, JAK1, NF1, NTRK1*, *BRAF* fusion gene but not in the *MEN1* gene, as well [9]. There are some reports on rare *MEN1* gene mutations, however, in patients with the undifferentiated sarcoma [10]. Our patient is a good example that MEN1 patients require intensive and lifelong surveillance, not only for typical endocrine but also for the non-endocrine and other uncommon tumors. One must be aware of the fact that there is generally no obvious genotype-phenotype correlation of specific mutations with the associated tumor varieties even among family members who carry the same mutation [3]. Moreover, this case of a novel *MEN1* gene mutation and atypical tumor such as fibromyxoid sarcoma raises the question if there will ever be a genotype-phenotype correlation as in other MEN syndromes. This case highlights the need for close cooperation between endocrinologists and oncologists due to the possibility of the occurrence of unusual cancers in this endocrine syndrome.

## Data Availability

The datasets generated and analysed during the current study are available in the GenBank database, accession number NM_130799 and ENSEMBL database, accession number ENST00000312049. Pathogenicity of identified mutation was verified in reference databases for mutations related to MEN1 syndrome (http://www.umd.be/MEN1/). The prediction of the new variant pathogenicity was performed with Mutation Taster (http://www.mutationtaster.org) and PROVEAN (http://provean.jcvi.org/index.php) software.

## Abbreviations

MEN1: Multiple endocrine neoplasia type 1
NET: Neuroendocrine tumor
